# Analysis of Fluorescent Carbon Nanodots Synthesized from Spices Through Thermal Processes Treatment

**DOI:** 10.3390/nano15080625

**Published:** 2025-04-19

**Authors:** David Semsey, Duyen H. H. Nguyen, Gréta Törős, Vivien Papp, János Pénzes, Tamás Vida, Áron Béni, Mahendra Rai, József Prokisch

**Affiliations:** 1 Institute of Animal Science, Biotechnology and Nature Conservation, Faculty of Agricultural and Food Sciences and Environmental Management, University of Debrecen, Böszörményi Street 138, 4032 Debrecen, Hungary; semi@gmail.hu (D.S.); toros.greta@agr.unideb.hu (G.T.); papp.vivien@agr.unideb.hu (V.P.); penzes.janos@agr.unideb.hu (J.P.); vida.levente@agr.unideb.hu (T.V.); mahendrarai7@gmail.com (M.R.); jprokisch@agr.unideb.hu (J.P.); 2 Doctoral School of Nutrition and Food Science, University of Debrecen, 4032 Debrecen, Hungary; 3 Institute of Life Sciences, Vietnam Academy of Science and Technology, 9/621 Vo Nguyen Giap Street, Linh Trung Ward, Ho Chi Minh City 70800, Vietnam; 4 Doctoral School of Animal Husbandry, University of Debrecen, Böszörményi Street 138, 4032 Debrecen, Hungary; 5 Institute of Agricultural Chemistry and Soil Science, Faculty of Agricultural and Food Sciences and Environmental Management, University of Debrecen, 138 Böszörményi Street, 4032 Debrecen, Hungary; beniaron@agr.unideb.hu; 6 Nanobiotechnology Laboratory, Department of Biotechnology, Sant Gadge Baba Amravati University, Amravati 444602, Maharashtra, India

**Keywords:** carbon dots, foods, cell integrity, Maillard reaction, heat treatment

## Abstract

Spices contain abundant essential oils and active compounds, which can be difficult to introduce into living cells due to their apolar, lipophilic nature. Carbon nanoparticles, produced through the Maillard reaction during food heat treatment, are small enough to enter cells easily. This study explores how thermal processing affects the formation of carbon nanodots (CNDs) in spices, revealing that higher temperatures boost CND synthesis, thus enhancing bioavailability and biological effectiveness. Interestingly, turmeric and black pepper enriched with CNDs notably influenced yeast fermentation, with an overall increase in antioxidant capacity, especially in turmeric and chili pepper. However, excessive heat occasionally reduced antioxidant activity, suggesting the breakdown of sensitive compounds. These findings highlight the potential of CND-enriched spices for health and nutrition applications.

## 1. Introduction

**Figure 1:**
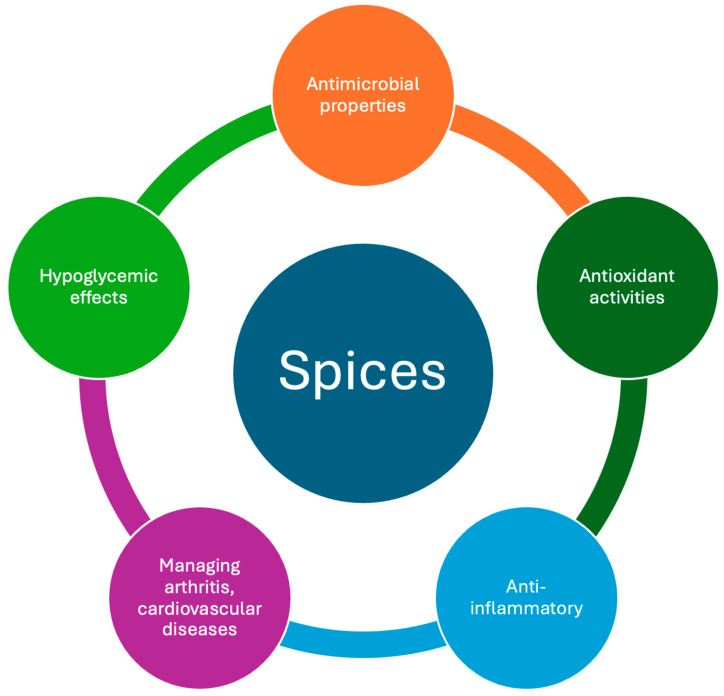
A schematic diagram illustrates the benefits of spices.

Spices, derived from various parts of plants such as seeds, fruits, roots, bark, and leaves, have been integral to human civilization for millennia. They serve as flavoring agents and possess significant medicinal properties, contributing to their extensive use in culinary and therapeutic practices. The chemical profile of spices is complex, encompassing a broad spectrum of bioactive compounds. Essential oils, alkaloids, phenolic compounds, flavonoids, and terpenes are among the primary constituents responsible for the distinctive aromas, flavors, and therapeutic effects of spices. For instance, capsaicin in chili peppers imparts pungency and exhibits analgesic properties, while curcumin in turmeric is renowned for its anti-inflammatory and antioxidant activities. Spices demonstrate a spectrum of biological activities attributed to their phytochemical content [1,2], illustrated in Figure 1.

Antimicrobial properties are evident in spices such as garlic (*Allium sativum*) and cinnamon (*Cinnamomum verum*), which have demonstrated efficacy against a variety of pathogens. Antioxidant activities, essential for combating oxidative stress and associated diseases, are notably high in spices like cloves (*Syzygium aromaticum*) and oregano (*Origanum vulgare*). Moreover, spices such as fenugreek (*Trigonella foenum-graecum*) and ginger (*Zingiber officinale*) have been studied for their anti-inflammatory and hypoglycemic effects, respectively [1,3]. The antimicrobial properties of spices make them valuable in food preservation. Compounds such as eugenol, thymol, and cinnamaldehyde inhibit the growth of spoilage microorganisms and pathogens, thus prolonging the freshness of food products. This traditional use aligns with modern scientific findings, supporting the integration of spices in contemporary food preservation strategies. The therapeutic potential of spices is vast, with applications ranging from traditional medicine to modern pharmacology. Turmeric, rich in curcumin, is extensively studied for its role in managing arthritis, cardiovascular diseases, and cancer. Similarly, black pepper (*Piper nigrum*), containing piperine, enhances the bioavailability of various drugs and nutrients, illustrating the synergistic potential of spice constituents in therapeutic formulations [3,4].

Spices are frequently used in fried and baked foods, during which the spices undergo heat treatment [5,6]. Recently, the food safety concerns regarding CNDs were considered [7], resulting in the necessity for research on the occurrence of CNDs in spices. Earlier research has shown that heat treatment can lead to the formation of carbon nanodots. (CNDs), likely through complex Maillard reaction mechanisms [8,9], where amino acids react with reducing sugars under heat, generating fluorescent nanoparticles with a carbon core and sizes typically below 10 nm [10,11,12]. As a result, spices used in fried and baked foods are also presumed to contain relatively high concentrations of CNDs due to their exposure to extreme heat conditions. These complex, primarily carbon-based particles have demonstrated a marked increase in scientific interest, attributed to their exceptional properties, including intense fluorescence, remarkable stability, the bioactive nature of their solid-state compounds, and high water solubility, whereas most active compounds in spices are fat-soluble, making them difficult for cells to absorb and utilize [4,13,14,15]. This work is based on the hypothesis that the active compounds in heat-treated, CND-enriched spices are more readily utilized by cells due to the presence of CNDs. The research aims to enhance the concentration of CNDs through targeted heat treatment of spices and to demonstrate that the active compounds in these treated spices are more bioavailable compared to those in “raw” spices.

## 2. Materials and Methods

### 2.1. Materials

All the ingredients were food-grade standards for the spice samples, including the following:

- Icing sugar (70 µm)–AGRANA Zucker GmbH (Wien, Austria);
- Ground black pepper–Alpaka Kft. (Szeged, Hungary);
- Ground turmeric–Alpaka Kft. (Szeged, Hungary);
- Ground chili pepper–Alpaka Kft. (Szeged, Hungary);
- Ground ginger–Alpaka Kft. (Szeged, Hungary);
- Ground clove–Alpaka Kft. (Szeged, Hungary);
- Cysteine powder–Vital Trend Kft. (Budapest, Hungary);
- Dried baker’s yeast (König Units Kft., Balmazújváros, Hungary).

The chemical excipients for the FRAP assay are as follows:

- Sodium-acetate-Spektrum 3D Kft. (Debrecen, Hungary);
- FeCl_3_•6H_2_O-VWR Chemicals BDH Kft. (Debrecen, Hungary);
- 2,4,6-tripyridyl-s-triazine–Sigma-Aldrich Kft. (Budapest, Hungary);
- Ascorbic acid-Molar Chemicals Kft. (Halásztelek, Hungary).

### 2.2. Methods

The experimental design consisted of four phases, beginning with sample preparation, followed by yeast foaming tests. Subsequently, the quantity of CNDs present in the heat-treated spice samples was measured using a Spectrofluorometer FP-8500, manufactured by Jasco, United States. In the final phase, the particle size of these nanodots was determined via an HPLC (ECOM Co., Praha, Czech Republic) with size exclusion column (SEC) linked with a fluorescent detector (FD).

#### 2.2.1. Spice Sample Preparation

**Figure 2:**
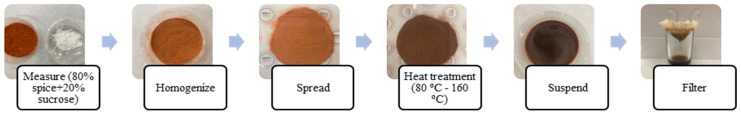
Flowchart of spice sample preparation; the extraction of CNDs in aqueous solution.

CNDs are linked to the Maillard reaction, which is enhanced in the presence of reducing sugars [16], and the spices were homogenized with icing sugar in an 80−20 ratio (80% spice, 20% powdered sugar with an average particle size of 70 microns). The mixtures were then spread evenly on a silicone sheet to a thickness of 3 mm and subjected to heat treatment at various temperatures for 1 h in a MEMMERT UF 60 drying chamber. As a control, a cysteine-icing sugar mixture was prepared in an 80−20 ratio (80% cysteine, 20% powdered sucrose with an average particle size of 70 microns), and heat-treated alongside the spice mixtures. The temperatures were increased in 20 °C increments from 80 °C to 160 °C. All experiments took place at Felföldi Confectionery Ltd. in Debrecen Hungary. The sample preparation process is well presented in Figure 2.

**Figure 3:**
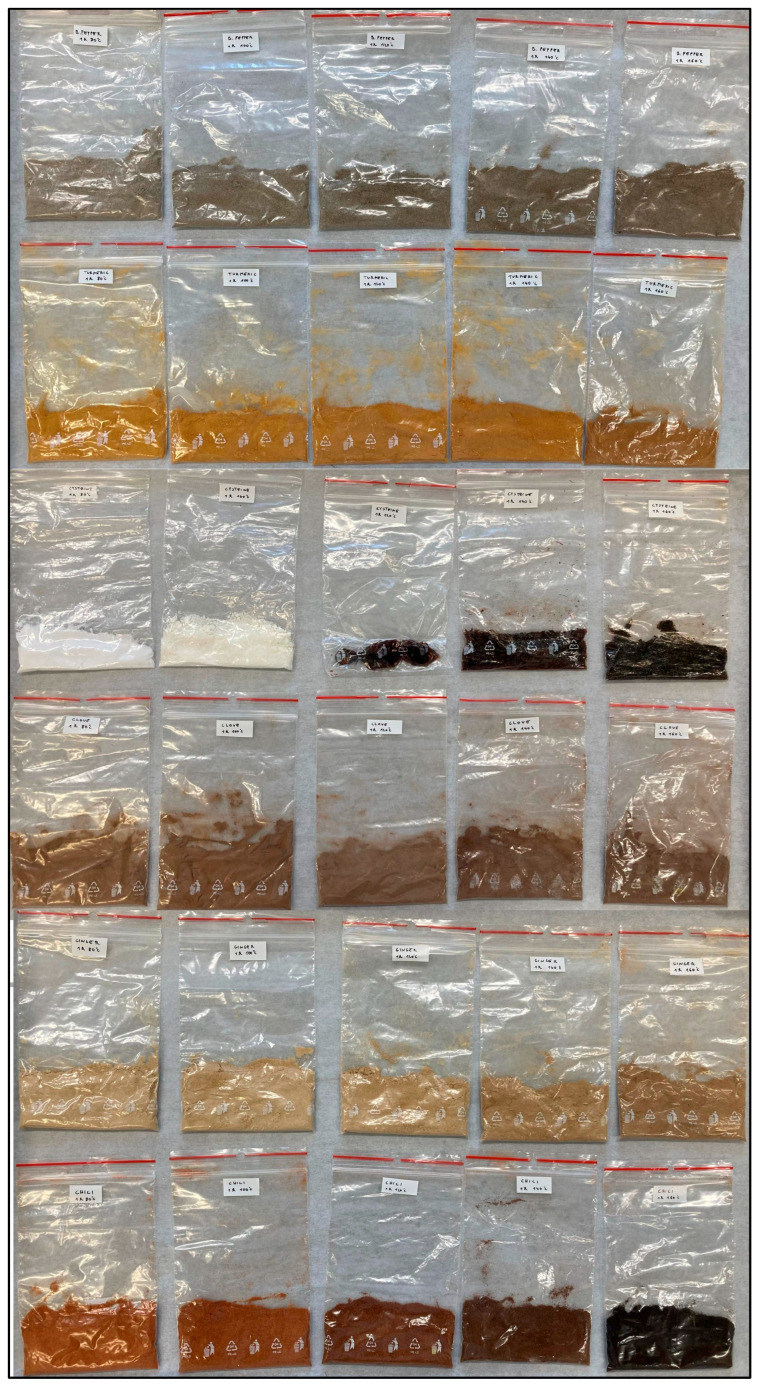
Color changes in powdered sugar-spice blends after 1 h heat treatments at various temperatures. From left to right: 80 °C, 100 °C, 120 °C, 140 °C, 160 °C. From top to bottom: black pepper, turmeric, cysteine, clove, ginger, chili.

The impact of heat treatment was visibly evident on the spices, particularly in the case of ground chili pepper, with noticeable changes observed across all samples above 140 °C. The cysteine control sample exhibited the most striking change, with the powder mixture changing color from yellow to black at temperatures exceeding 100 °C. The different color variations in the samples as an effect of heat treatment are well illustrated in Figure 3.

#### 2.2.2. Yeast Tests

Yeast has served as a model organism in research for nearly 70 years, with its industrial and scientific relevance driving advancements in toxicity testing [17,18]. Cell count assays are among the simplest methods for evaluating the growth-inhibitory effects of toxic agents. They offer advantages such as speed and cost-effectiveness through microscopic cell number determination. However, modifications like cell fixation are necessary to ensure consistent and reliable results. These assays are particularly valuable for regulatory decision-making, where clear and timely data presentation is crucial. Simple assays that focus only on particle–cell interactions without assessing overall cell viability are limited in scope. While growth inhibition tests can effectively evaluate yeast toxicity, they may not fully capture the effects of nanoparticles on specific cell lines. Given nanoparticles’ unique properties and potential hormonal disruptions, it is recommended to include such assays within broader panels to ensure comprehensive validation and hazard assessment [18,19].

The production of carbon dioxide by yeast is a crucial indicator of both its viability and metabolic activity. This process serves as a reliable measure of the yeast’s fermentation efficiency and its ability to perform essential metabolic functions. Generally, higher carbon dioxide production is associated with more active and viable yeast. During fermentation, yeast releases carbon dioxide as a byproduct of its breakdown of sugars, which are converted into alcohol and carbon dioxide. This complex fermentation process is integral to various industries, including baking, brewing, and winemaking, where yeast is central in producing desired flavors, textures, and alcohol content that meet customer expectations. Monitoring yeast’s carbon dioxide production provides producers with valuable insights into the health and performance of their yeast cultures, ensuring optimal results in the final product. Understanding and managing yeast’s carbon dioxide production is an essential skill in yeast biology, microbiology, and fermentation science [20,21,22].

**Figure 4:**
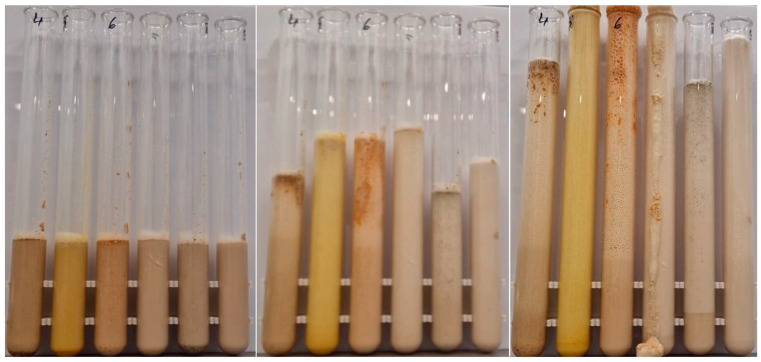
Yeast suspension foaming tests. From left to right: 2% ground clove, 2% ground turmeric, 2% ground chili pepper, 2% ground ginger, 2% ground black pepper, spice-free control. From left to right: at the 1st minute of the test, at the 5th minute of the test, at the 10th minute of the test.

Since one objective of this study was to investigate how accumulated CNDs in heat-treated spices affect live cells and whether there are differences compared to untreated spices, initially a special series of experiments with sucrose-yeast suspensions was conducted. A novel, unique rapid method has been developed that provides a representative and comparable assessment of yeast fermentation activity and its variations within 10 min. For this, 6.25 g of dried baker’s yeast (*Saccharomyces cerevisiae*) was mixed with 6.25 g of crystalline sucrose, followed by homogenization with 36.5 g of tap water at 30 °C. Then, 1 g of each heat-treated spice blend was added to the suspension, and yeast activity and foam height were monitored over 10 min in 300 mm tall glass test tubes, as clearly illustrated in Figure 4. The time required for the suspension meniscus to reach the top of the test tube was compared, and the meniscus levels were monitored at the final moment of measurement. The initial suspension level was 110 mm in all cases. The foaming tests were conducted in 3 repetitions.

#### 2.2.3. FRAP Tests

We quantitatively evaluated the antioxidant capacities of the chosen spices using the Ferric Reducing Antioxidant Power (FRAP) method assay. For sample preparation, 1 g of each spice was accurately weighed into centrifuge tubes, followed by adding 40 mL of distilled water. The samples underwent ultrasonic extraction for 30 min using an Olympus Endosonic ultrasonic cleaner (T1711, 2098). Subsequently, the extracts were filtered and centrifuged at 5000 rpm for 10 min using a Hettich-EBA 21 centrifuge. The resulting supernatant was then collected and utilized for further analytical procedures.

In accordance with the protocol set by Benzie and Strain [23], the FRAP reagent was prepared by combining 25 mL of sodium acetate buffer at pH 3.6 with 10 mL of 20 mM of FeCl_3_•6H_2_O and 10 mL of 10 mM 2,4,6-tripyridyl-s-triazine (TPTZ). For the assay, 10 µL of the extract was blended with 65 µL of distilled water and 2250 µL of FRAP reagent. Distilled water was used as the blank instead of the extract. The mixture was incubated at room temperature for 8 min, after which absorbance was measured at 593 nm using a UV-160A spectrophotometer (Shimadzu, Kyoto, Japan). A standard 5.67 mM ascorbic acid solution was used to create the calibration curve. The results were reported as milligrams of ascorbic acid for each gram of dry weight (mg AA/g DW). The measurements were conducted in three repetitions.

#### 2.2.4. Spectrofluorometer Tests

The extraction of carbon nanodots (CNDs) from the prepared samples followed previously published methodologies, incorporating specific modifications to optimize the process [21]. Initially, the spice samples were homogenized in ion-exchange water to form a suspension with a concentration of 5 mass percent (m/m%). To achieve the isolation of CNDs, the resulting suspension was subjected to a purification process that involved passing it through a cellulose-based column. It was followed by filtration through an analytical-grade filter paper with a pore size of 0.22 µm to remove residual particulates. After the purification step, the fluorescence characteristics of the extracted solutions were analyzed using a Jasco FP-8500 spectrofluorometer manufactured by Jasco International Co., Ltd. (Osaka, Japan). Fluorescence measurements were conducted at an excitation wavelength of 370 nm, under the previously established and modified protocols [16].

#### 2.2.5. HPLC Tests

Size exclusion chromatography (SEC) with a fluorescence detector was employed to accurately assess the particle size distribution and molar mass of the isolated CNDs, following the modified method of Shafigulina et al. (2019) [24]. The analysis was performed using a high-performance liquid chromatography (HPLC) system (Ecom Spol. Sr.o., Praha, Czech Republic), configured with the following optimized parameters: (1) a mobile phase composed of a mixture of 20% acetonitrile and 80% water to ensure efficient separation; (2) a constant flow rate maintained at 0.7 mL/min to optimize resolution; (3) an injection volume set at 5 μL to facilitate precise quantification; (4) a Shimadzu RF-20A fluorescence detector for sensitive detection of the CNDs; and (5) fluorescence measurement set with an excitation wavelength of 380 nm and an emission wavelength of 460 nm. The selection of this mobile phase composition was specifically intended to minimize hydrophobic interactions within the chromatographic column, thereby enhancing the accuracy and reproducibility of the separation process [25]. Additionally, the detector settings were meticulously fine-tuned according to the 3D fluorescence spectra of the CNDs to ensure optimal signal capture and analytical sensitivity. Agilent AdvanceBio SEC 300A, 2.7 µm, 4.6 × 300 mm column was used for this measurement.

Statistical analyses were performed utilizing SPSS software (version 29.0.0). An Analysis of Variance (ANOVA) was employed for the comparative evaluation of normally distributed datasets, whereas the Kruskal–Wallis test was applied to datasets exhibiting non-normal distribution. Statistical significance was determined at a threshold of *p* < 0.05. All graphical representations were generated using Microsoft Excel 365 Edition.

## 3. Results

### 3.1. Characterization of CNDs in Spices

**Figure 5:**
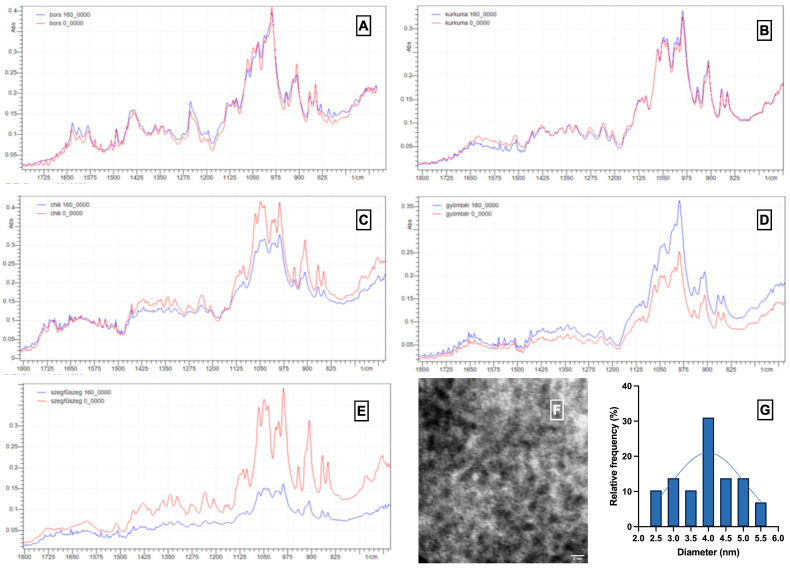
Characterization of carbon nanodots (CNDs) in spices. (**A**–**E**) FTIR analysis of spices including (**A**) pepper, (**B**) turmeric, (**C**) chili pepper, (**D**) ginger, and (**E**) clove, with red and blue indicating the results before and after heat treatment. (**F**) TEM images of CNDs extracted from red chili pepper, displayed with a 5 nm scale bar. (**G**) Size distribution of CNDs in the heat-treated clove sample.

Figure 5 presents a characterization of carbon nanodots (CNDs) extracted from various spices, focusing on their structural and morphological properties. Figure 5A–E illustrate the Fourier Transform Infrared (FTIR) spectra of pepper, turmeric, chili pepper, ginger, and clove, respectively. The comparison of spectra before (red lines) and after (blue lines) heat treatment reveals significant changes in the functional groups present. First, significant changes were observed in the IR spectra following heating, indicating chemical transformations associated with CNDs formation. All samples exhibited increased absorbance within the 1000–1300 cm^−1^ range, which suggests enhanced C–O and C–N stretching vibrations commonly linked to carboxylic acids, alcohols, and amines—functional groups typically present on the surface of CNDs [26]. Furthermore, the appearance or intensification of bands near 1650–1725 cm^−1^ implies the formation of C=O bonds, likely from aldehydes, ketones, or amides generated during the carbonization of organic precursors.

However, trends can be observed in the degree of change across different spices. For example, clove (Figure 5E) and chili pepper (Figure 5C) exhibit more pronounced increases in peak intensities post-heating, especially in the fingerprint region, which suggests higher levels of functionalization. This is consistent with their high polyphenol and essential oil content, which are known to decompose into smaller aromatic and oxygenated structures that facilitate the nucleation of CNDs. In contrast, turmeric (Figure 5B) and ginger (Figure 5D) show subtler changes, indicating a less intense transformation or lower yield of CNDs under the same heating conditions. Turmeric, rich in curcuminoids, may follow a different degradation pathway without CNDs formation as effectively as eugenol-rich clove or capsaicin-rich chili.

These spectral variations indicate that the chemical composition of the initial material plays a crucial role in determining the type and quantity of functional groups present after CND formation. The increased presence of oxygen- and nitrogen-containing functionalities following heating supports the hypothesis that surface passivation occurs naturally during the thermal process, thereby enhancing CND solubility and optical activity.

Figure 5F shows a Transmission Electron Microscopy (TEM) image of CNDs from red chili pepper post-heat treatment. It reveals well-dispersed, quasi-spherical nanoparticles with a 5 nm scale bar, confirming their nanoscale dimensions. Figure 5G shows the size distribution of CNDs in the heat-treated red chili pepper sample, represented by a histogram and Gaussian fit. Most CNDs range from 2 to 5 nm, peaking at 3.5 nm. The combined FTIR and TEM data suggest that the heat treatment method effectively transforms the spice components into CNDs with specific surface functional groups and controlled size distribution.

### 3.2. Yeast Tests

**Figure 6:**
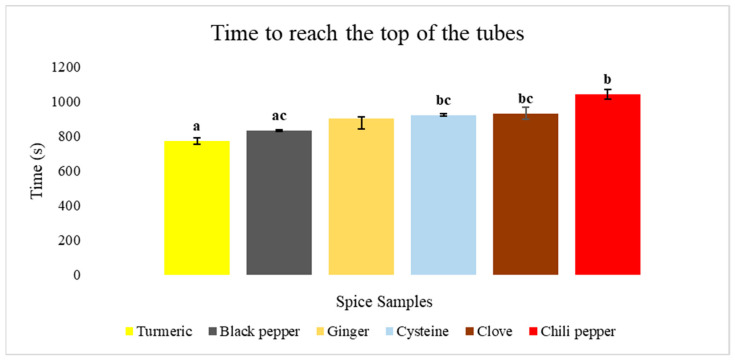
The average rise times of the spiced yeast suspensions from the initial level (110 mm) to the maximum level (300 mm) in the test tubes, expressed in seconds. Distinct letters (a–c) indicate a significant difference at a 95% confidence interval.

**Figure 7:**
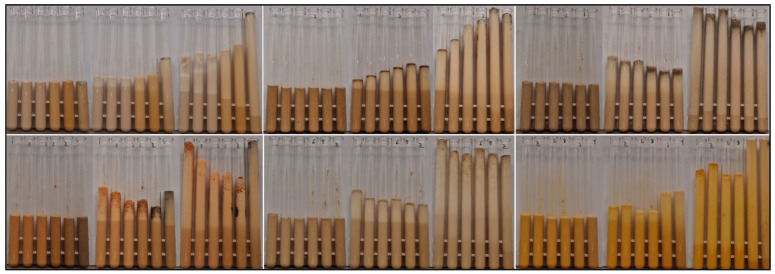
The levels of spiced yeast suspensions in test tubes at the end of the measurement cycle, expressed in millimeter.

During the yeast foaming tests, the rising rate of spiced yeast suspensions in test tubes—representing the fermentation reaction—was fastest for turmeric and black pepper, while it was slowest for chili. Black pepper and ginger exhibited the highest average rise rates per spice type and also the highest suspension levels per test tube. These results are clearly illustrated in Figure 6 and Figure 7.

Based on non-normally distributed data, the Kruskal–Wallis statistical analysis showed significant differences in the time required to reach the peak of various spices (*p* = 0.011). Specifically, turmeric exhibited the shortest time to reach the peak, requiring 771.7 ± 11.7 s, which was significantly less (*p* < 0.05) than cysteine (921.7 ± 16.9 s), clove (930.0 ± 20.2 s), and chili (1040 ± 35.0 s) with considerable amounts under 0.001. On the other hand, chili required significantly more time than black pepper (831.7 ± 4.4 s), with a *p*-value of 0.006.

**Figure 8:**
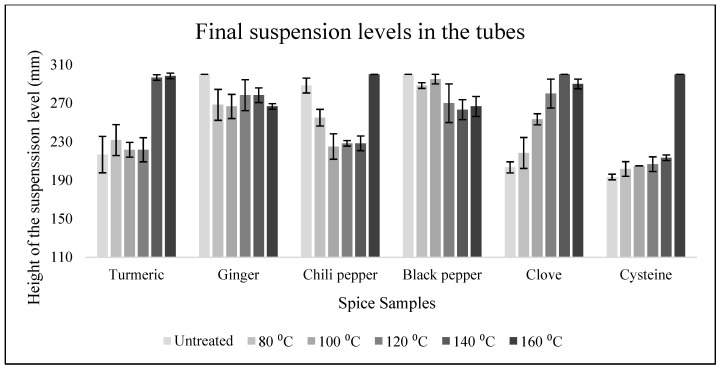
Yeast suspension foaming tests. Upper row from left to right: 2% cysteine (untreated, 80 °C, 100 °C, 120 °C, 140 °C, 160 °C), 2% ground clove (untreated, 80 °C, 100 °C, 120 °C, 140 °C, 160 °C), 2% ground black pepper (untreated, 80 °C, 100 °C, 120 °C, 140 °C, 160 °C). Lower row from left to right: 2% ground chili pepper (untreated, 80 °C, 100 °C, 120 °C, 140 °C, 160 °C), 2% ground ginger (untreated, 80 °C, 100 °C, 120 °C, 140 °C, 160 °C), 2% ground turmeric (untreated, 80 °C, 100 °C, 120 °C, 140 °C, 160 °C).

The different spices exhibited distinct effects on the yeast suspension, each displaying unique foaming characteristics. For example, turmeric induced an exceptionally rapid fermentation reaction, yet the average foam height at the end of the cycle was lower compared to other spices. Based on Figure 8, it can also be observed, with 1–2 exceptions, that higher heat treatments at 140–160 °C enhanced yeast activity.

### 3.3. FRAP Tests

**Figure 9:**
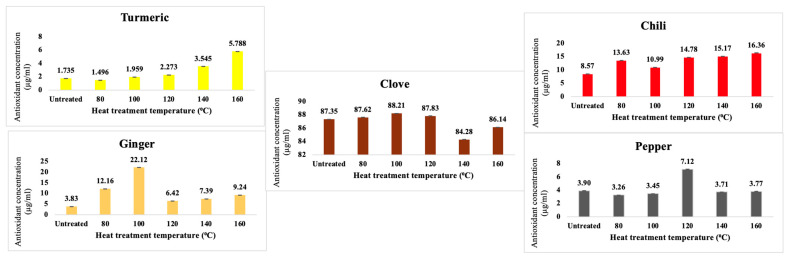
Antioxidant activities of untreated and heat-treated spice samples.

For most spices, an increase in heat treatment temperature generally led to an enhancement in antioxidant activity, with a few exceptions. However, in the case of pepper, ginger, and clove, a decline in activity was observed above 100–120 °C. Chili exhibited the highest antioxidant activity, while turmeric showed the most significant increase, as clearly illustrated in Figure 9.

The spices exhibited significant differences in antioxidant activity relative to each other. However, it was also observed within the set that the values did not follow a normal distribution due to thermal processing at different temperatures, especially in the case of turmeric.

### 3.4. CNDs Content

The fluorescence intensities of the heat-treated spice sample solutions, measured at 370 nm excitation, varied significantly depending on the type of spice and the applied temperature, with intensity maxima ranging from 333 to 8576 a.u. The cysteine control exhibited the highest fluorescence, reaching up to 11,565 a.u., indicating its high reactivity and efficiency as a precursor for carbon nanodots (CNDs) formation.

In general, increasing the temperature promoted greater fluorescence intensity, supporting the hypothesis that elevated thermal energy facilitates the formation of CNDs. However, in several spice samples—such as clove, turmeric, and black pepper—the fluorescence peaked at intermediate temperatures (e.g., 120 °C or 140 °C) and decreased slightly at 160 °C. This trend may reflect a balance between enhanced carbonization at moderate temperatures and partial degradation or aggregation at excessive heating, which can quench fluorescence.

Among the tested spices, red chili pepper and ginger showed strong fluorescence responses, suggesting favorable precursor composition (e.g., amino acids, sugars, or phenolic compounds) for CNDs formation. In contrast, turmeric and black pepper produced the lowest intensities (e.g., <600 a.u.), possibly due to a lower carbon yield or poor fluorescence quantum yield of the generated CNDs. Notably, the fluorescence enhancement was most evident above 120 °C, marking a critical threshold temperature for CNDs synthesis in these matrices.

**Figure 10:**
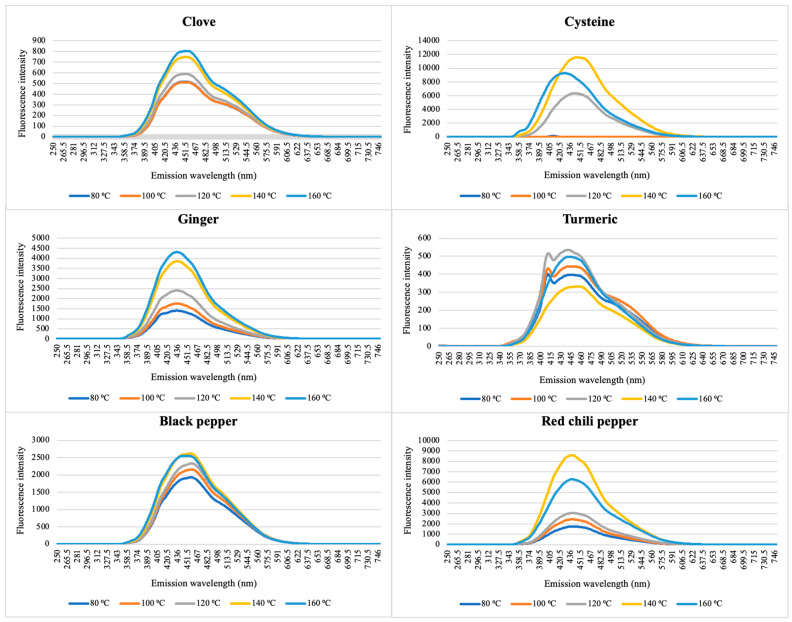
Fluorescent spectra of CNDs extracted from heat-treated spices at 370 nm excitation.

**Figure 11:**
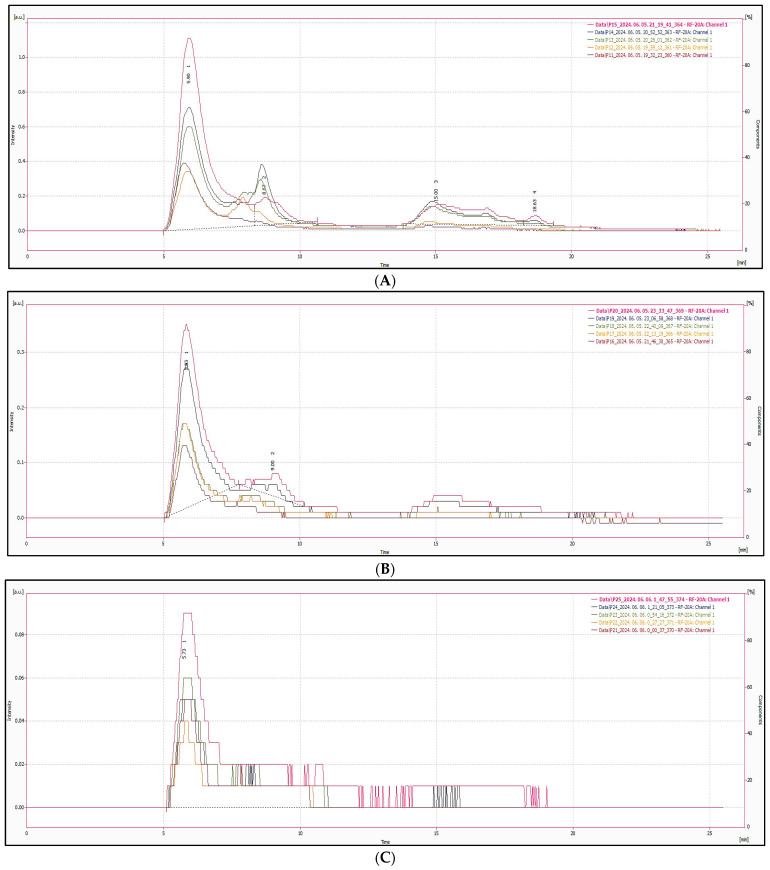
Chromatogram of heat-treated samples (Part 1), from top to bottom at 160 °C, 140 °C, 120 °C, 100 °C, 80 °C, including (**A**) red chilis, (**B**) turmeric, and (**C**) clove.

**Figure 12:**
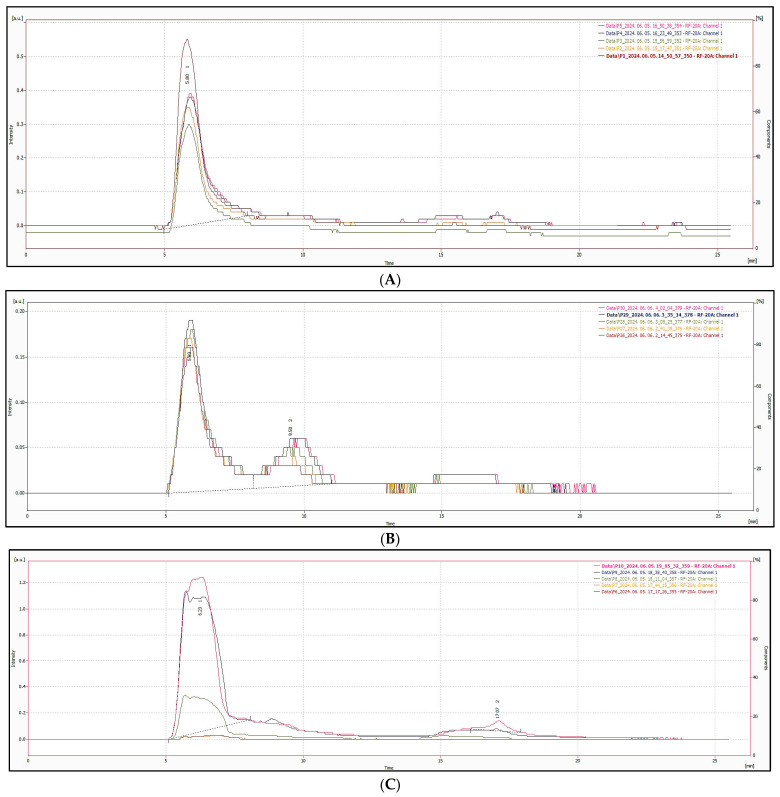
Chromatogram of heat-treated samples (Part 2), from top to bottom at 160 °C, 140 °C, 120 °C, 100 °C, 80 °C, including (**A**) ginger, (**B**) black pepper and (**C**) cysteine.

The emission spectra in Figure 10 show consistent broad peaks centered around 430–440 nm across samples, characteristic of blue-emitting CNDs. These spectral features suggest that the observed fluorescence arises from similar surface states or emissive traps, although intensity differences reflect variability in yield and structure of the synthesized CNDs. Figure 11 and Figure 12 show the HPLC-SEC-FD chromatogram of heated spices. Figure 11 displays the chromatogram of (A) red chilis, (B) turmeric, and (C) clove, while Figure 12 presents the chromatogram of (A) ginger, (B) black pepper, and (C) cysteine. The CNDs of heated spices were collected as the initial peak in HPLC-SEC-FD, with an estimated size of less than 5 nm based on molecular weight calculations.

### 3.5. CND Molar Mass and Size

**Figure 13:**
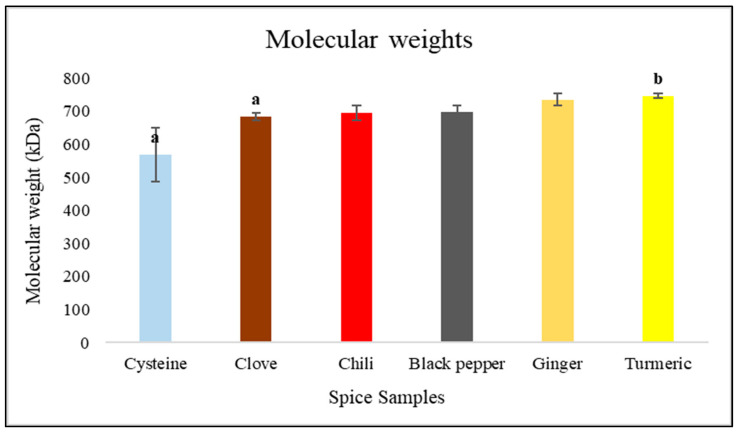
Average molecular weights of carbon seed nanodots formed in heat treated spice samples. Distinct letters (a–b) indicate a significant difference at a 95% confidence interval.

The average molecular weights of the CNDs measured by size-exclusion chromatography are shown in Figure 13.

Kruskal–Wallis statistical analysis revealed significant differences in the non-normal distributed data with molecular weight (kDa) of various spices (*p* < 0.001). The molecular weight of turmeric (746.5 ± 16.49 kDa) was significantly higher (*p* < 0.05) than that of clove (683.34 ± 12.16 kDa) and cysteine (568.670 ± 81.240 kDa). The molecular weights of chili (694.96 ± 21.21 kDa), black pepper (698.4 ± 18.68 kDa), and ginger (735.7 ± 39.01 kDa) did not differ significantly from those of the other spices (*p* > 0.05).

## 4. Discussion

The complex interrelationship between thermal processing and the consequent formation of CNDs within spices unveils a compelling facet of bioactive enhancement that necessitates comprehensive investigation. This study systematically elucidates the role of elevated temperatures in facilitating the synthesis of CNDs, thereby substantially augmenting the bioavailability and biological functionality of these highly valued culinary components. Fluorescence intensity measurements reveal a distinct correlation, demonstrating that increased thermal exposure proportionally enhances CND production, a trend particularly pronounced in chili and ginger. This finding aligns with previous studies suggesting that the thermal degradation of organic compounds catalyzes the MR, yielding fluorescent nanomaterials with unique physicochemical properties that may confer multiple functional advantages [8,10,12,16].

The impact of CND formation on yeast fermentation dynamics was particularly notable, as spices enriched with thermally induced CNDs exhibited substantial modulation of yeast metabolism. Turmeric and black pepper, in particular, elicited the most pronounced fermentation responses, suggesting that CNDs may enhance yeast activity—potentially through improved sugar transport mechanisms or the modulation of key cellular interactions essential for metabolic processes. Conversely, despite exhibiting the highest fluorescence intensity, chili pepper demonstrated a relatively attenuated fermentation rate. This phenomenon may be attributed to its capsaicin content, which, owing to its well-documented antimicrobial properties, could inhibit specific metabolic pathways. Collectively, these findings provide a refined perspective on the interplay between CND synthesis, bioavailability enhancement, and the intrinsic chemical properties of spices, which, in turn, shape their biological implications in the domains of health and nutrition.

Assessments of antioxidant capacity, conducted via FRAP assays, further substantiate the premise that CNDs enhance the functional attributes of various spices. The majority of tested spices exhibited increased antioxidant activity following heat treatment, with turmeric and chili pepper displaying the most pronounced improvements. This observation suggests that CND formation may not only preserve but also potentiate antioxidant functionality, potentially through the stabilization of phenolic compounds or the enhancement of radical scavenging mechanisms that safeguard cellular integrity against oxidative stress. However, it is noteworthy that excessive thermal exposure occasionally resulted in diminished antioxidant capacity, indicating the degradation of heat-sensitive bioactive compounds crucial for maintaining health benefits. Chromatographic analysis of the synthesized CNDs, with a few exceptions, revealed a consistent molecular size and distribution across the different spice matrices examined, suggesting a uniform MR pathway irrespective of spice composition. This consistency underscores the potential for the deliberate production of CND-enriched spice extracts, which may hold significant applications in both culinary and pharmaceutical contexts, offering novel avenues for health optimization.

## 5. Conclusions

In conclusion, this study highlights the dual role of thermal processing as a transformative factor in spice bioactivity, facilitating bioavailability via CND synthesis while simultaneously modulating antioxidant potential and enhancing yeast fermentation efficiency. Future research should focus on elucidating the precise molecular mechanisms underlying CND interactions with cellular structures and their broader implications for human health. Additionally, optimizing thermal treatment parameters to maximize beneficial outcomes while mitigating the degradation of vital bioactive compounds will be essential for advancing innovations in food science and nutraceutical development.

## Data Availability

The raw data supporting the conclusions of this article will be made available by the authors on request.
